# The Correlation between Oral Lichen Planus and Thyroid Pathologies: A Retrospective Study in a Sample of Italian Population

**DOI:** 10.1055/s-0043-1772247

**Published:** 2023-09-20

**Authors:** Sara Piloni, Francesco Ferragina, Ida Barca, Elvis Kallaverja, Maria Giulia Cristofaro

**Affiliations:** 1 Department of Neurosciences, Reproductive and Odontostomatological Sciences, Maxillofacial Surgery Unit, University Federico II, Naples, Italy; 2 Department of Experimental and Clinical Medicine, Maxillofacial Surgery Unit, “Magna Graecia” University, Catanzaro, Italy

**Keywords:** oral lichen planus, thyroid diseases, autoimmune diseases

## Abstract

**Objectives** The association between oral lichen planus and thyroid disorders, especially hypothyroidism and Hashimoto's thyroiditis, has been discussed in current literature with conflicting outcomes.

**Materials and Methods** The study retrospectively evaluated the thyroid status in patients diagnosed with oral lichen planus and oral lichenoid lesions. A case–control approach was used to prove that thyroid disorders were statistically significant risk factors for oral lichen planus and oral lichenoid lesions.

**Statistical Analysis** To evaluate these associations, odds ratios (ORs) were used. ORs precision and statistical significance were estimated using a 95% confidence interval (CI) and *p* -value, respectively.

**Results** A total of 307 patients were involved in the study: 158 females and 149 males. OR, 95% CIs, and *p* -values were analyzed. Patients with thyroid diseases showed an increased risk of developing oral lichen planus (OR: 4.29, 95% CI: 1.85–9.96, *p* -value: 0.0007) and oral lichenoid lesions (OR: 2.76, 95% CI: 1.24–6.13, *p* -value: 0.0129). This association was maintained in patients with oral lichen planus, while also considering hypothyroidism (OR: 3.74, 95% CI: 1.46–9.58, *p* -value: 0.0059) and Hashimoto's thyroiditis (OR: 4.57, 95% CI: 1.58–13.23, *p* -value: 0.005) alone. The correlation of hypertension, diabetes, dyslipidemia, and smoking status with oral lichen planus and oral lichenoid lesions was also evaluated but no statistical significance was found.

**Conclusion** Even if further investigations are needed, the association between oral lichen planus and oral lichenoid lesions with thyroid pathologies should be taken into consideration by endocrinologists due to the potential malignancy of these disorders.

## Introduction

Oral lichen planus (OLP) is a common immune-mediated mucocutaneous disorder, usually described in women (male: female ratio 1:2), between the fifth and sixth decade. Worldwide its estimated prevalence was reported to be between 0.22 and 5%, with a mean value of 1 to 2%. It can be accompanied by lesions of other mucous membranes or the skin in about 20 to 60% of affected patients.

The OLP clinical forms are usually classified as white lichen, which included papular, reticular, and plaque forms; red lichen, which included all atrophic or erosive forms, regardless of the concurrent presence of the white form. 1 2 3 It was hypothesized that these different clinical manifestations may be related to dissimilar biological events. Moreover, the significant involvement of specific classes of T lymphocytes, responsible for marked tissue damage, could determine the development of erosive lesions. 4

There are also other immune reactions with similar clinical or histopathological manifestations called oral lichenoid lesions (OLL), usually unilateral and related to some drugs (nonsteroidal anti-inflammatory drugs [NSAIDs] and beta-blockers) or oral mucosa exposure to immune restorations such as amalgam.

To date, the mechanisms underlying the development of OLP are not yet well known. There is, though, a common agreement in the scientific community that it is caused by an immune-mediated process. Several studies showed an association of OLP with the presence of lymphocyte infiltrate of CD4 + and CD8 + lymphocytes at the level of the connective epithelial interface and functional polymorphisms in the regulatory regions of the genes coding for the proinflammatory cytokines tumor necrosis factor-alpha (TNF-α), interleukin-12 (IL-12), IL-18, and chemokines CXCL9 and CXCL10. 5 6 7 8

Apart from immune disorders, other factors, such as dental restorations, heredity, infections, and stress, assume importance in a minority of patients. For example, it was observed in different studies that anxiety and stress are very common in OLP patients. 9 OLP exacerbations seemed associated with an increase in psychological stress, but no significant association has been proven yet. A possible connection lies in the fact that acute and chronic psychological stress can induce changes in the innate and adaptive immune response. 10

In consideration of a probable auto-immunological cause of OLP and OLL, various associations between this disease and other systemic conditions were found in various populations in numerous studies. Among these conditions, the most studied association is between OLP and OLL with thyroid disorders (TD), especially with autoimmune TD. In the last decade, some authors reported a statistically significant association between OLP and hypothyroidism 11 12 13 or Hashimoto's thyroiditis (HT), 14 meanwhile, others disproved it. 15

No definite consensus was reached yet. Most studies only involved populations from other continents (especially Asia) and most studies involved endocrinology departments.

The exact etiology remains unknown, although immune dysregulation appears to play a critical role in OLP development and progression, which seemed to result from the interaction between the immune system and a broad range of extrinsic and self-antigens. 16

In such a discordant context, the authors, through this retrospective study, conducted in a sample of Italian population referring to the U.O.C. of Maxillofacial Surgery of the “Magna Graecia” University, intended to evaluate the association of OLP and OLL (diagnosed by biopsy) with TD, hypothyroidism and HT in particular, using a case–control approach, comparing OLP and OLL patients (case 1 and 2 groups) with patients who resulted negative for known oral pathologies and only presented aspecific inflammatory infiltrates (control group).

## Materials and Methods

This retrospective comparative study was conducted on a cohort of 307 patients who were referred to the U.O.C. of Maxillofacial Surgery of the “Magna Graecia” University (Catanzaro, Italy) from January 2010 to December 2020. The study population was retrospectively selected by analyzing the medical records of patients investigated for mucosal lesions of the oral cavity.

The treatment of patients was in line with the Declaration of Helsinki 2013 and each patient signed informed consent. The study was approved by the Research Ethics Committee of the “Magna Graecia” University of Catanzaro.

The inclusion criteria of the study were as follows: patients over 18 years old; histopathological diagnosis of OLP, OLL, or negative for known oral pathologies.

The exclusion criteria of the study were as follows: pregnant or breastfeeding women; patients with complicated systemic pathologies; patients with acute infections; patients who refused histopathological examination; patients who refused to sign the informed consent.

Patients were divided into three groups: patients with the diagnosis of OLP (case group 1), OLL (case group 2), and negative for oral pathologies (NOP) (control group).

The analyzed lesions were found in different locations: lips, labial commissure, oral vestibule, cheek mucosa, tongue, oral pelvis, alveolar crest, retromolar trine, palate, tuber maxillae, and tonsils.

The final diagnosis of OLP and OLL was based on diagnostic criteria, clinical and histopathologic, proposed by van der Meij et al. 17 Clinical criteria were the presence of bilateral lesions and reticular, erosive, atrophic, bullous and plaque type patterns. Histopathologic criteria were bands of cellular infiltration, especially lymphocytes, in the superficial part of connective tissue, liquefaction degeneration in the basal cell layer, no evidence of epithelial dysplasia. The diagnosis of OLP was made when a clinical suspicion of OLP was confirmed by positive histopathological criteria. OLL was diagnosed when a clinical suspicion of OLP was not confirmed at the histopathological examination.

Each patient was individually and retrospectively traced for further anamnesis on his general health status, focusing on pre-existing or co-existing systemic diseases, smoking, and daily medication intake. Cardiovascular diseases (especially arterial hypertension), metabolic pathologies (diabetes and dyslipidemia), immuno-rheumatological diseases (rheumatoid arthritis, for example), neoplastic pathologies (oral tumors and others), and TD were carefully investigated. Specific TD types were recorded, including hypothyroidism, hyperthyroidism, thyroid nodules, HT, and Graves-Basedow's disease. Regarding TD, hormone replacement therapy intake (levothyroxine) and positivity to antithyroid antibodies were also considered.

The association between OLP, OLL, and NOP patients with TD was then estimated and compared with the association between OLP, OLL, and NOP patients with other diseases and risk factors.

### Statistical Analysis

The study had a case–control approach. Due to its retrospective nature, to evaluate the association between exposure to a supposed risk factor (TD and other pathologies) and the outcome of OLP and OLL, odds ratios (ORs) were used. OR measures the odds of a certain outcome (OLP and OLL) with or without exposure to a certain factor (TD and other pathologies). OR is calculated by dividing the odds of exposure among cases (outcome) divided by the odds of exposure among controls (no outcome; Table 1 and Formula 1 ).

**Table 1:** Odds ratio table

	Outcome	No outcome	
Exposure	a	b	a + b
No exposure	c	d	c + d
	a + c	b + d	

**Formula 1** Odd ratio calculation.

Odd ratio = odds of exposition among cases/odds of exposure among controls = (a / c) / (b / d) = a x d / b x c

When OR is equal to 1, exposure does not affect the odds of the outcome. When OR is higher than 1, exposure determines higher odds of outcome; meanwhile, when OR is lower than 1, exposure determines lower odds of the outcome.

The precision of the OR was then estimated using a 95% confidence interval (CI). Large 95% CIs report lower precision of the OR; meanwhile, small 95% CIs have higher precision, but they do not indicate statistical significance. A two tailed *p* -value is used to measure statistical significance: if the *p* -value is lower than 0.05, results are statistically significant.

All statistical analyses were performed using MedCalc Software Ltd, Acacialaan 22, 8400 Ostend, Belgium (2022).

## Results

Clinical records of 307 patients, who were referred to the U.O.C. of Maxillofacial Surgery of the “Magna Graecia” University, were examined. A total of 158 (51.47%) females and 149 (48.53%) males were enrolled in the retrospective study. Seventy-six patients (24.76%) were affected by OLP, 36 (47.37%) females and 40 (52.63%) males, with a mean age of 63 years (range: 49–75). Although OLP was clinically observed on any oral cavity site, the most involved one was the cheek mucosa (42, 55.26%). One hundred and thirty-three patients (43.32%) were affected by OLL, 72 (54.14%) females and 61 (45.86%) males, with a mean age of 59 years (range: 20–91). The site most affected by OLL was also the cheek mucosa (56 patients, 42.11%). Among the 98 control group patients (31.92%), 48 (48.98%) patients were females and 50 (51.02%) were males, with a mean age of 55 years (range: 20–81). The site most affected by nonspecific inflammatory infiltrates negative for known oral pathologies was also the cheek mucosa (47 patients, 47.96%; Table 2 ).

**Table 2:** Demographic data and patients characteristics

Patients	OLP (case 1 group)	OLL (case 2 group)	NOP (control group)
Total	76	133	98
Males	40 (52.63%)	61 (45.86%)	48 (48.98%)
Females	36 (47.37%)	72 (54.14%)	50 (51.02%)
Lips	0	2 (1.50%)	3 (3.06%)
Labial commissure	3 (3.95%)	5 (3.76%)	4 (4.08%)
Oral vestibule	1 (1.32%)	1 (0.75%)	0
Cheek mucosa	42 (55.26%)	56 (42.11%)	47 (47.96%)
Tongue	15 (19.74%)	33 (24.81%)	26 (26.53%)
Oral pelvis	2 (2.63%)	3 (2.26%)	1 (1.02%)
Alveolar crest	3 (3.95%)	6 (4.51%)	5 (5.1%)
Retromolar trine	5 (6.58%)	10 (7.52%)	5 (5.1%)
Palate	3 (3.95%)	14 (10.53%)	6 (6.12%)
Tuber maxillae	2 (2.63%)	2 (1.50%)	1 (1.02%)
Tonsils	0	1 (0.75%)	0

Abbreviations: NOP, negative for oral pathologies; OLL, oral lichenoid lesion; OLP, Oral lichen planus.

The most common type of OLP lesions was the reticular type (48, 63.16%), followed by atrophic (11, 14.47%), erosive (11, 14.47%), plaque (5, 6.58%), and bullous (1, 1.32%; Table 3 ). Among all 307 patients, skin lichen planus was encountered in only two female patients with OLL.

**Table 3:** OLP subtypes

OLP	76
Atrophic	11 (14.47%)
Erosive	11 (14.47%)
Plaque	5 (6.58%)
Bullous	1 (1.32%)
Reticular	48 (63.16%)

Abbreviation: OLP, Oral lichen planus.

As shown in Table 4 , a history of any TD was found in 23 (30.26%) of the patients with OLP: 17 (22.37%) had hypothyroidism, the most representative disorder, 15 (19.74%) of which presented HT; 3 patients (3.06%) had hyperthyroidism, 2 (2.63%) of which were affected by Graves-Basedow's disease; 5 (6.58%) patients had thyroid nodules (2 of which concomitant with hypothyroidism).

**Table 4:** Patients' comorbidities and risk factors

Patients	OLP (case group 1)	OLL (case group 2)	NOP (control group)
Total	76	133	98
Males	40 (52.63%)	61 (45.86%)	48 (48.98%)
Females	36 (47.37%)	72 (54.14%)	50 (51.02%)
Thyroid pathologies	23 (30.26%)	29 (21.8%)	9 (9.18%)
Hypothyroidism	17 (22.37%)	19 (14.29%)	7 (7.14%)
Hyperthyroidism	3 (3.95%)	2 (1.50%)	1 (1.02%)
Thyroid nodules	5 (6.58%)	11 (8.27%)	3 (3.06%)
Hashimoto's thyroiditis	15 (19.74%)	15 (11.28%)	5 (5.1%)
Graves-Basedow's disease	2 (2.63%)	1 (0.75%)	0
Cardiovascular pathologies	37 (48.68%)	59 (44.36%)	47 (47.95%)
Hypertension	36 (47.37%)	44 (33.08%)	45 (45.91%)
Metabolic pathologies	17 (22.37%)	30 (22.56%)	24 (24.49%)
Diabetes	13 (17.11%)	21 (15.79%)	9 (9.18%)
Dyslipidemia	11 (14.47%)	15 (11.28%)	18 (18.37%)
Immuno-rheumatologic pathologies	2 (2.63%)	12 (9.02%)	3 (3.06%)
Neoplastic pathologies	14 (18.42%)	25 (18.8%)	7 (7.14%)
Oral cancer	9 (11.84%)	25 (18.8%)	5 (5.1%)
Other cancers	6 (7.9%)	2 (1.50%)	2 (2.04%)
Smoke	30 (39.47%)	44 (33.08%)	29 (29.59%)

Abbreviations: NOP, negative for oral pathologies; OLL, oral lichenoid lesion; OLP, oral lichen planus.

In the OLL group, TD were found in 29 patients (21.8%): 19 (14.29%) had hypothyroidism, the most common disorder also among OLL patients, 15 (11.28%) of which presented HT; 2 patients (1.50%) had hyperthyroidism, 1 (0.75%) of which affected by Graves-Basedow's disease; 11 patients (8.27%) had thyroid nodules (3 of which presented hypothyroidism as well).

In the NOP group, TD were encountered in 9 patients (9.18%): 7 (7.15%) had hypothyroidism, 5 (5.1%) of which had HT; 1 patient (1.02%) was affected by hyperthyroidism; 3 patients (3.06%) presented thyroid nodules (2 of which in combination with hypothyroidism).

The use of thyroxine-based drugs was reported in 15 patients (19.74%) with OLP, 17 patients (12.78%) with OLL, and 5 patients (5.1%) NOP. The 3 patients (0.98%) with Graves-Basedow's disease (2 OLP, 1 OLL) underwent total thyroidectomy. Furthermore, the positivity for antithyroglobulin antibodies (TGA) was found in all 35 patients (11.4%) with a diagnosis of HT; meanwhile, the other 8 hypothyroid patients (2.6%) presented negative TGA, and 7 of them showed thyroid nodules at the same time ( Table 4 ).

As shown in Table 5 , TD, hypothyroidism and HT in particular, showed to be statistically significant risk factors for OLP (OR: 4.29, 95% CI: 1.85–9.96, *p* -value: 0.0007; OR: 3.74, 95% CI: 1.46–9.58, *p* -value: 0.0059; OR: 4.57, 95% CI: 1.58–13.23, *p* -value: 0.005, respectively). TD in general seemed to be a statistically relevant risk factor also for OLL (OR: 2.76, 95% CI: 1.24–6.13, *p* -value: 0,0129). This statistically significant association with OLL was not proven for hypothyroidism and HT, studied separately (OR: 2.17, 95% CI: 0.87–5.38, *p* -value: 0.0965; OR: 2.36, 95% CI: 0.83–6.74, *p* -value: 0.1075, respectively). Therefore, OLP showed a stronger correlation with TD than OLL.

**Table 5:** Differences in TD, hypothyroidism, HT, hypertension, diabetes, dyslipidemia, and smoke positivity for OLP and OLL patients and negative control cases

	Odds ratio	95% CI	*p* -Value
Patients with OLP vs. patients with NOP (76 vs. 98) Negative TD (53 vs. 89) Positive TD (23 vs. 9)	4.29	1.85–9.96	0.0007
Patients with OLP vs. patients with NOP (76 vs. 98) Negative hypothyroidism (59 vs. 91) Positive hypothyroidism (17 vs. 7)	3.74	1.46–9.58	0.0059
Patients with OLP vs. patients with NOP (76 vs. 98) Negative HT (61 vs. 93) Positive HT (15 vs. 5)	4.57	1.58–13.23	0.005
Patients with OLP vs. patients with NOP (76 vs. 98) Negative hypertension (40 vs. 53) Positive hypertension (36 vs. 45)	1.06	0.58–1.93	0.8492
Patients with OLP vs. patients with NOP (76 vs. 98) Negative diabetes (63 vs. 89) Positive diabetes (13 vs. 9)	2.04	0.82–5.06	0.1241
Patients with OLP vs. patients with NOP (76 vs. 98) Negative dyslipidemia (65 vs. 80) Positive dyslipidemia (11 vs. 18)	0.75	0.33–1.71	0.4951
Patients with OLP vs. patients with NOP (76 vs. 98) No smoking (46 vs. 69) Smoking (30 vs. 29)	1.55	0.82–2.92	0.1732
Patients with OLL vs. patients with NOP (133 vs. 98) Negative TD (104 vs. 89) Positive TD (29 vs. 9)	2.76	1.24–6.13	0.0129
Patients with OLL vs. patients with NOP (133 vs. 98) Negative hypothyroidism (114 vs. 91) Positive hypothyroidism (19 vs. 7)	2.17	0.87–5.38	0.0965
Patients with OLL vs. patients with NOP (133 vs. 98) Negative HT (118 vs. 93) Positive HT (15 vs. 5)	2.36	0.83–6.74	0.1075
Patients with OLL vs. patients with NOP (133 vs. 98) Negative hypertension (89 vs. 53) Positive hypertension (44 vs. 45)	0.58	0.34–1	0.0484
Patients with OLL vs. patients with NOP (133 vs. 98) Negative diabetes (112 vs. 89) Positive diabetes (21 vs. 9)	1.85	0.81–4.25	0.1443
Patients with OLL vs. patients with NOP (133 vs. 98) Negative dyslipidemia (118 vs. 80) Positive dyslipidemia (15 vs. 18)	0.57	0.27–1.19	0.1313
Patients with OLL vs. patients with NOP (133 vs. 98) No smoking (89 vs. 69) Smoking (44 vs. 29)	1.18	0.67–2.07	0.5729

Abbreviations: CI, confidence interval; HT, Hashimoto's thyroiditis; NOP, negative for oral pathologies; OLL, oral lichenoid lesion; OLP, oral lichen planus; TD, thyroid disorders.

All the other variables analyzed (hypertension, diabetes, dyslipidemia, and smoking) did not show a statistically significant impact on OLP and OLL. Only a minor protective value was found for hypertension for OLL (OR: 0.58, 95% CI: 0.34–1, *p* -value: 0.0484; Table 5 ).

## Discussion

The hypothesis of an association between OLP and TD was born from reports of patients, affected by TD, that discovered OLP lesions as well. It was taken into consideration in many studies, but results were controversial for several factors.

The lack of uniformity in the design and methodology of these studies could provide a partial explanation for this dispute. Furthermore, OLP patients were not always diagnosed by a specialist and the diagnosis of TD was sometimes based on self-reported data by patients but, above all, the clinical feedback of these injuries was not only systematically confirmed by histological examination but also limited to clinical suspicious cases. 15

An association between OLP and concomitant autoimmune diseases, such as HT, can although be assumed, due to immuno-mediated pathogenesis of OLP.

Numerous data come from the Chinese population, where a greater prevalence of TD, in particular HT, was found in patients with OLP 18 19 20 and the correlation between these conditions seemed to reside in common autoimmune pathways. 19

Current worldwide literature has shown that, among TD, hypothyroidism is the most frequently found condition in patients with a diagnosis of OLP. Arduino et al, for example, in one of the widest prospective studies on the topic, conducted on a population of Northern Italy, highlighted that patients with TD were associated with an almost three times greater probability of having OLP. 1 This could be because hypothyroidism, especially the subclinical one, is the most frequent diagnosis among individuals with TD.

To date, however, there is still no final thesis to explain the coexistence of OLP and TD. Lo Muzio et al have recently suspected a causal or predisposing role of HT in patients with OLP, suggesting that the circulating thyroid antibodies could help trigger an organ-specific autoimmune response also in the oral mucosa, leading to the development of OLP lesions 14 19 21 22

In our study, a positivity for TGA was found in all 35 patients (11.4%) diagnosed with HT; meanwhile, the other 8 hypothyroid patients (2.6%) presented negative TGA, and 7 of them showed thyroid nodules at the same time.

Nodular thyroid diseases do not have autoimmune etiology. However, immunological changes have been detected in this condition as well: such as an increase in the number of dendritic cells and circulating lymphocytes, and the production of inflammatory mediators, such as IL-6 and TNF-α, also involved in the pathogenesis of the OLP. 1

Several other studies have found no significant association between OLP and TD, including hypothyroidism and HT, but this data was derived from retrospective studies in which OLP diagnosis was not always carried out histologically. 23 24 25 In addition, in a study conducted in an Iranian court, beyond the lack of histological confirmation, also genetic and ethnic idiosyncrasies may have played a role in the results. 15

Due to the unevenness of the current literature and uncertain etiology, 26 27 the Authors wanted to verify the association between OLP and TD in a population of Southern Italy. In our retrospective case-control study, clinical suspicion of OLP, OLL, and aspecific inflammatory infiltrate was confirmed in every patient with histopathological confirmation. We proved that, among the variables taken into consideration, only TD, especially hypothyroidism and HT, could be possible risk factors for the onset of OLP, showing statistical significance (OR: 4.29, 95% CI: 1.85–9.96, *p* -value: 0.0007; OR: 3.74, 95% CI: 1.46–9.58, *p* -value: 0.0059; OR: 4.57, 95% CI: 1.58–13.23, *p* -value: 0.005, respectively). The same statistical significance, although less important, was demonstrated in the OLL group only for TD (OR: 2.76, 95% CI: 1.24–6.13, *p* -value: 0,0129), but not for hypothyroidism and HT analyzed separately (OR: 2.17, 95% CI: 0.87–5.38, *p* -value: 0.0965; OR: 2.36, 95% CI: 0.83–6.74, *p* -value: 0.1075, respectively). Therefore, OLP showed a stronger correlation with TD than OLL.

In line with our findings, Siponen et al assessed the association of any TD with the incidence of OLP and OLL and confirmed a stronger association between any TD with OLP, than with OLL. This correlation was proven for hypothyroidism not only for OLP, as in our study, but also for OLL, conversely with our study. 13

All the other variables analyzed (hypertension, diabetes, dyslipidemia, and smoking) did not show a statistically significant impact on OLP and OLL. 28

In some studies, exogenous factors such as smoking and alcohol consumption seemed to have a protective effect, reducing the risk of TD. Specifically, smoking seemed to lower anti-thyroid peroxidase and TGA levels. Garcia-Pola et al, for example, reported a lower frequency of smokers and alcohol drinkers among patients with OLP, compared with controls. 11 This difference in our study was not encountered and the only possible protective factor was hypertension and only for OLL (OR: 0.58; 95% CI: 0.34–1; *p* -value: 0.0484).

Robledo-Sierra et al reported significantly higher consumption of thyroid drugs (levothyroxine) and NSAIDs in patients with OLP and proved a significant relationship between levothyroxine intake and OLP. 12 The same results were also reported in our study, in which the use of thyroxine-based drugs was found in 15 patients (19.74%) with OLP, 17 patients (12.78%) with OLL, and 5 patients (5.1%) NOP.

In this study, there were some limitations. Its retrospective nature did not allow adequate control and measurement of the different variables and the use of an to collect information from patients may have introduced observer and response biases. In addition, this study was carried out on a relatively geographically limited population; therefore, a generalization of the results to other populations must be done with caution. Despite recent studies reporting similar results, studies with larger and multicenter samples are needed to affirm the actual cause-and-effect link between TD and OLP.

## Conclusion

In our retrospective case–control study in a sample of Italian population, we established that, among the variables taken into consideration, only TD, in particular hypothyroidism and HT, can be considered risk factors for the onset of OLP, confirming the importance of investigating OLP in patients with endocrinological and immunological diseases. Due to the retrospective nature of this study, the presence of some incorrect or missing data is possible, especially regarding drug intake and TD diagnosis. Undoubtedly, future research and more precise data collection are required.

TD should be better assessed through the measurement of the serum level of thyroid hormones and autoantibodies and clinical suspicion of OLL and OLP should be always confirmed histologically. Furthermore, the involvement of larger samples of patients can reduce confusion factors.

In conclusion, considering the possible link between OLP and thyroid/immunological diseases and the potential malignancy of OLP, it would be necessary for endocrinologists and general practitioners to pay attention to this possible association, as OLP is a pathology that requires regular and careful follow-up over time.
